# Corneal Biomechanical Properties in Varying Severities of Myopia

**DOI:** 10.3389/fbioe.2020.595330

**Published:** 2021-01-21

**Authors:** Mohammad-Reza Sedaghat, Hamed Momeni-Moghaddam, Abbas Azimi, Zohreh Fakhimi, Mohammed Ziaei, Zeynad Danesh, Cynthia J. Roberts, Naeemeh Monfared, Alireza Jamali

**Affiliations:** ^1^Eye Research Center, Mashhad University of Medical Sciences, Mashhad, Iran; ^2^Health Promotion Research Center, Zahedan University of Medical Sciences, Zahedan, Iran; ^3^Department of Optometry, School of Paramedical Sciences, Mashhad University of Medical Sciences, Mashhad, Iran; ^4^Refractive Errors Research Center, Mashhad University of Medical Sciences, Mashhad, Iran; ^5^Department of Ophthalmology, New Zealand National Eye Centre, University of Auckland, Auckland, New Zealand; ^6^Ophthalmology & Visual Science, Biomedical Engineering, The Ohio State University, Columbus, OH, United States; ^7^Department of Optometry, School of Rehabilitation Sciences, Iran University of Medical Sciences, Tehran, Iran

**Keywords:** myopia, cornea, corneal biomechanics, CorVis ST, ORA, axial length

## Abstract

**Purpose:** To investigate corneal biomechanical response parameters in varying degrees of myopia and their correlation with corneal geometrical parameters and axial length.

**Methods:** In this prospective cross-sectional study, 172 eyes of 172 subjects, the severity degree of myopia was categorized into mild, moderate, severe, and extreme myopia. Cycloplegic refraction, corneal tomography using Pentacam HR, corneal biomechanical assessment using Corvis ST and Ocular Response Analyser (ORA), and ocular biometry using IOLMaster 700 were performed for all subjects. A general linear model was used to compare biomechanical parameters in various degrees of myopia, while central corneal thickness (CCT) and biomechanically corrected intraocular pressure (bIOP) were considered as covariates. Multiple linear regression was used to investigate the relationship between corneal biomechanical parameters with spherical equivalent (SE), axial length (AXL), bIOP, mean keratometry (Mean KR), and CCT.

**Results:** Corneal biomechanical parameters assessed by Corvis ST that showed significant differences among the groups were second applanation length (AL2, *p* = 0.035), highest concavity radius (HCR, *p* < 0.001), deformation amplitude (DA, *p* < 0.001), peak distance (PD, *p* = 0.022), integrated inverse radius (IR, *p* < 0.001) and DA ratio (DAR, *p* = 0.004), while there were no significant differences in the means of pressure-derived parameters of ORA between groups. Multiple regression analysis showed all parameters of Corvis ST have significant relationships with level of myopia (SE, AXL, Mean KR), except AL1 and AL2. Significant biomechanical parameters showed progressive reduction in corneal stiffness with increasing myopia (either with greater negative SE or greater AXL), independent of IOP and CCT. Also, corneal hysteresis (CH) or ability to dissipate energy from the ORA decreased with increasing level of myopia.

**Conclusions:** Dynamic corneal response assessed by Corvis ST shows evidence of biomechanical changes consistent with decreasing stiffness with increasing levels of myopia in multiple parameters. The strongest correlations were with highest concavity parameters where the sclera influence is maximal.

## Introduction

Myopia is the most common eye disorder in the world with a worldwide prevalence of more than 22% (Wu et al., 2015). High myopia can increase the risk of ocular problems such as glaucoma, retinal detachment, and chorioretinal degeneration (Paluru et al., 2003).

The cornea is a viscoelastic tissue and corneal biomechanics, including the material properties of the cornea, determine its shape. Biomechanical properties of the cornea are influenced by extracellular matrix components, collagen lamella organization, osmotic pressure, hormonal factors, and environmental conditions (Pinero and Alcon, 2015). In recent years a wide variety of work has been completed on corneal biomechanics in health and disease with the introduction of non-invasive devices able to measure *in vivo* biomechanical parameters (Perez-Rico et al., 2015; Lee et al., 2016b; Vinciguerra et al., 2016). A better understanding of corneal biomechanical response can allow for better diagnosis and staging of various corneal disorders, refinement of suitable patients for refractive surgery or intrastromal ring segment implantation and provide further insights into biomechanics-modulating treatments such as corneal crosslinking (CXL) and keratoplasty (Kling and Hafezi, 2017; Ziaei et al., 2019, 2020). However, the analysis and evaluation of corneal biomechanics is complex as the cornea is a viscoelastic tissue.

In recent years a wide variety of work has been completed on corneal biomechanics in health and disease with the introduction of non-invasive devices able to measure *in* vivo biomechanical deformation response such as the Ocular Response Analyzer (ORA) and Corvis ST (Lee et al., 2016b; Vinciguerra et al., 2016).

Corneal hysteresis (CH) and corneal resistance factor (CRF) which are the main biomechanical parameters for evaluating corneal viscoelasticity are measured by the ORA (Reichert Ophthalmic Instruments, Buffalo, NY, USA) (Roberts, 2016). Previous studies have reported a significantly lower CH in high myopia compared to emmetropia (Shen et al., 2008). Assessment of corneal biomechanical response using the ORA in a Chinese population confirmed the reduction of CH only in high myopia, while spherical equivalents had a positive correlation with both CH and CRF (Jiang et al., 2011). A similar study in a Caucasian population reported slightly lower values of CH in high myopes, with no correlation between CRF and refractive error. This suggest that minor alteration of corneal viscoelastic properties occur in moderate myopia (Plakitsi et al., 2011).

Corvis ST (OCULUS Optikgeräte GmbH; Wetzlar, Germany) is a newly introduced device for measuring biomechanical deformation response of the cornea. Lee et al. compared the corneal biomechanical parameters in myopic and emmetropic subjects using the Corvis ST and reported greater corneal mean outward applanation velocity in high myopic subjects compared to emmetropes (Lee et al., 2016a). They also reported a positive correlation for deformation amplitude (DA) with axial length (AXL); and a negative correlation for highest concavity radius (HCR) with mean keratometry reading and AXL (Lee et al., 2016a).

Although a number of studies have evaluated corneal biomechanical parameters in mild to moderate myopia (Shen et al., 2008; Chang et al., 2010; Xu et al., 2010; Jiang et al., 2011; Bueno-Gimeno et al., 2014; Wang et al., 2015; Lee et al., 2016a; Qiu et al., 2016) there is a paucity of research on extreme myopia (myopia >9D). Also, control of the effects of corneal thickness and intraocular pressure as two influencing factors on corneal biomechanics are important points that are sometimes ignored in comparative studies. Therefore, the present prospective study was designed to assess the biomechanical parameters of the cornea in different amounts of myopia matched according to corneal thickness, corneal curvature, and intraocular pressure. In addition, the correlation of biomechanical parameters with spherical equivalent, IOP, AXL, corneal curvature, and thickness was assessed.

## Materials and Methods

This cross-sectional study was conducted from November 2019 to January 2020 at a tertiary referral center in Iran. One hundred and seventy two eyes of 172 myopic patients seeking refractive surgery were recruited. The study was approved by the Ethics Committee of Mashhad University of Medical Sciences (Code No.: 980275). Written, informed consent was obtained from all patients after they voiced understanding of the purpose and the procedures of the study in accordance with the Declaration of Helsinki.

Inclusion criteria were myopic spherical equivalent (SE) with refractive astigmatism lower than 1.5 diopters (D). Subjects with a history of ocular pathology such as glaucoma, cataract, ocular hypertension, corneal ectasia, or prior refractive surgery or those with history of contact lens wear in the last 3 months, systemic disease such as diabetes, hypertension, and collagen-vascular disorders were excluded. To avoid the effect of diurnal variation on corneal biomechanics, all biomechanical measurements were performed between 4 and 8 pm.

### Patient Assessment

Along with detailed ophthalmic examinations including visual acuity, slit-lamp biomicroscopy and tonometey, cycloplegic refraction was done finally after application of Tropicamide 1% eye drops, administrated 3 times at 5 min intervals and after a 30 min waiting period. Auto-kerato-refractometer 8000 (Topcon Corporation, Tokyo, Japan), Pentacam HR (Oculus, Wetzlar, Germany), Ocular Response Analyser (ORA, Reichert Ophthalmic Instruments, Buffalo, NY, USA), Corvis ST (Oculus; Wetzlar, Germany), and IOLMaster 700 (Carl Zeiss Meditec, Jena, Germany) exams were performed for all subjects.

### Measurements Variables

The degree of myopia was classified into mild (-3.0D < SE ≤ −0.50D), moderate (-3.0D ≥SE>−6.0D), severe (-9.0D < SE ≤ −6.0D) and extreme (SE ≤ −9.0D) myopia (Tang et al., 2018). The tomographic parameters included in the study were mean anterior keratometry in the central 3 mm and central corneal thickness (CCT). Corneal biomechanical parameters derived by Corvis ST were applanation length at the first and second applanations (AL1 and AL2), the velocity at the first and second applanations (AV1 and AV2), peak distance (PD), highest concavity radius (HCR), deformation amplitude (DA), and non-compensated (IOPnct) and biomechanically corrected (bIOP) intra-ocular pressure. The novel parameters provided by Corvis ST such as stiffness parameter at the first applanation (SPA1), integrated inverse radius (IR), and deformation amplitude ratio (DA ratio) were also analyzed. The classic parameters by ORA were corneal hysteresis (CH) and corneal resistance factor (CRF). All imaging techniques were done by an experienced and qualified operator.

In the dynamic corneal response parameters and corneal tomography parameters evaluated using Corvis ST and Pentacam HR, only measurements with an “OK” quality specification were included in the analysis. The quality of the pressure-derived parameters by ORA was evaluated according to the waveform score (WS) provided by the device and scans with a WS of more than 3.5 were included in the analysis. The axial length provided by the IOLMaster 700 was repeated several times and the average of three repeated measurements with a difference of <0.02 mm were used.

### Statistical Analysis

Data were analyzed using SPSS.26 software (SPSS, Chicago, IL). The normality of data was checked using the Kolmogorov-Smirnov test. The Kruskal-Wallis test was used to compare axial length (AXL), corneal curvature (Mean KR), central corneal thickness (CCT), and IOP between the different myopic groups. A general linear model was used to compare corneal biomechanical parameters obtained using the ORA and Corvis ST in various degrees of myopia, while central corneal thickness (CCT) and biomechanically corrected intraocular pressure (bIOP) were considered as covariates. Pairwise comparisons were performed using Dunn-Bonferroni *post-hoc* test., Multiple linear regression with stepwise method of predictors (Entry *P* < 0.1; removal *P* > 0.2) was carried out to investigate the relationship between each corneal biomechanical parameter with SE, AXL, CCT, Mean KR, and bIOP. The significance level was set at a *p* < 0.05. Data from only one eye randomly selected from each participant were used for analysis.

## Results

The study comprised of 172 eyes of 172 myopic patients (80 males, 92 females). The subjects' mean age was 27.89 ± 4.51 years with a range of 20–35 years. Assessed eyes in mild, moderate, high and extreme myopia groups were 46 (26.7%), 46 (26.7%), 44 (25.6%), and 36 (20.9%) eyes, respectively. There was no significant difference in the mean age (*p* = 0.212) and the gender distribution among the different groups (*p* = 0.156).

The mean refractive status (sphere, cylinder, and spherical equivalent), axial length and mean KR, CCT, and the IOPs measured by Corvis ST in different myopia groups are presented in Table 1. There was no significant difference between the groups apart from AXL (*p* < 0.001) and refractive status (*p* < 0.001).

**Table 1:** Mean and SD of refraction, axial length, mean keratometry reading, intra-ocular pressure, and central corneal thickness separately in different myopia groups.

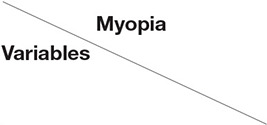	**Mean** **±** **SD (95% CI)**				***P*-value**
	**Low (*n =* 46)**	**Moderate (*n =* 46)**	**High (*n =* 44)**	**Extreme (*n =* 36)**	
Sphere (D)	−1.73 ± 0.37 (−1.84 to −1.62)	−3.66 ± 0.58 (−3.83 to −3.49)	−6.60 ± 0.81 (−6.84 to −6.35)	−11.53 ± 2.81 (−12.49 to −10.58)	<0.001*
Cylinder (D)	−0.40 ± 0.37 (−0.51 to−0.29)	−0.55 ± 0.40 (−0.67 to −0.44)	−0.92 ± 0.81 (−1.05 to −0.80)	−1.14 ± 0.47 (−1.30 to −0.98)	<0.001*
SE (D)	−1.93 ± 0.37 (−2.04 to −1.82)	−3.93 ± 0.58 (−4.11 to −3.76)	−7.06 ± 0.84 (−7.31 to −6.80)	−12.09 ± 2.80 (−13.04 to −11.14)	<0.001*
AXL (mm)	24.47 ± 0.69 (24.27–24.68)	24.86 ± 0.65 (24.67–25.06)	26.06 ± 0.84 (25.81–26.32)	27.79 ± 1.84 (27.10–28.47)	<0.001*
Mean KR (D)	43.58 ± 1.17 (43.23–43.93)	43.82 ± 1.43 (43.40–44.25)	44.17 ± 1.38 (43.75–44.59)	43.87 ± 1.39 (43.39–44.36)	0.093
IOPnct (mmHg)	15.59 ± 1.38 (15.42–16.00)	15.83 ± 1.37 (15.53–16.24)	16.13 ± 1.31 (15.72–16.53)	15.81 ± 1.28 (15.35–16.27)	0.102
bIOP (mmHg)	15.68 ± 1.35 (15.24–16.12)	15.80 ± 1.64 (15.29–16.30)	16.55 ± 0.97 (16.05–17.05)	16.10 ± 0.01 (16.10–16.10)	0.069
CCT (μm)	536.79 ± 31.75 (526.89–546.68)	537.54 ± 35.71 (526.94–548.15)	535.40 ± 26.12 (527.36–543.43)	529.85 ± 25.06 (521.11–539.85)	0.552

Significant results are represented with*. (n = 172 eyes) (SD, Standard Deviation; CI, Confidence Interval; SE, Spherical Equivalent; AXL, Axial Length; KR, Keratometry Reading; IOPnct, non–corrected Intra–Ocular Pressure; bIOP, Biomechanically corrected IOP; CCT, Central Corneal Thickness).

The mean corneal biomechanical parameters assessed using Corvis ST and ORA while CCT and bIOP were considered as covariates are presented in Table 2. Considering the Corvis's classic biomechanical parameters, there was a statistically significant difference for AL2 (8.043, *p* = 0.035), HCR (*p* < 0.001), DA (18.78 *p* < 0.001) and PD (*p* = 0.022) among the different myopic groups. Dunn-Bonferroni *post-hoc* test showed in Table 1. Among the new parameters provided by Corvis ST, IR (*p* < 0.001), and DAR (*p* = 0.004) showed a statistically significant difference among the various groups. Dunn-Bonferroni *post-hoc* test showed a significant difference in mean IR between all pairs except for low with moderate (*p* = 0.199) myopia groups, and in mean DAR between all pairs except for low with moderate (*p* = 0.632) and high with extreme (*p* = 0.693) myopia.

**Table 2:** Mean and SD corneal biomechanical parameters using Corvis ST and ORA in different myopia groups and CCT and bIOP as covariates.

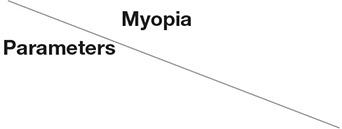	**Low (*n =* 46) (a)**	**Moderate (*n =* 46) (b)**	**High (*n =* 44) (c)**	**Extreme (*n =* 36) (d)**	***P*–value [Pairwise Comparisons]**
	**Mean ± SD (95% CI)**	**Mean ± SD (95% CI)**	**Mean ± SD (95% CI)**	**Mean ± SD (95% CI)**	
**Corvis ST parameters**					
AL1 (mm)	2.36 ± 0.62 (2.27–2.46)	2.31 ± 0.58 (2.22–2.40)	2.30 ± 0.60 (2.20–2.390)	2.23 ± 0.68 (2.12–2.33)	0.308
AL2 (mm)	1.73 ± 0.81 (1.61–1.86)	1.60 ± 0.85 (1.47–1.73)	1.50 ± 0.83 (1.38–1.63)	1.49 ± 0.94 (1.35–1.64)	0.035* [a,c:0.012, a,d: 0.013]
AV1 (m/s)	0.13 ± 0.03 (0.13–0.13)	0.13 ± 0.03 (0.13–0.14)	0.13 ± 0.03 (0.13–0.14)	0.14 ± 0.03 (0.13–0.14)	0.614
AV2 (m/s)	−0.33 ± 0.034 (−0.34 to −0.32)	−0.30 ± 0.03 (−0.33 to −0.27)	−0.32 ± 0.03 (−0.35 to −0.28)	−0.34 ± 0.03 (−0.36to−0.33)	0.223
HCR (mm)	7.72 ± 0.56 (7.56–7.90)	7.52 ± 0.66 (7.36–7.68)	7.24 ± 0.70 (7.02–7.35)	6.40 ± 0.49 (6.28–6.45)	<0.001* [a,b: 0.078, Other pairs: <0.05]
DA (mm)	1.03 ± 0.13 (1.01–1.05)	1.04 ± 0.12 (1.02–1.06)	1.10 ± 0.13 (1.09–1.12)	1.14 ± 0.14 (1.12–1.16)	<0.001* [a,b: 0.548, Other pairs: <0.05]
PD (mm)	5.10 ± 0.49 (5.02–5.17)	5.12 ± 0.46 (5.05–5.20)	5.26 ± 0.49 (5.18–5.33)	5.29 ± 0.54 (5.20–5.37)	0.022* [a,c:0.002, a,d: 0.001, b,c: 0.011, b,d: 0.005]
SPA1 (mmHg/mm)	106.03 ± 136.36 (85.06–127.0)	99.83 ± 129.89 (79.86–119.81)	123.83 ± 134.91 (102.60–143.55)	105.75 ± 151.45 (81.80–128.37)	0.439
IR (mm^−1^)	7.46 ± 1.25 (7.27–7.65)	7.63 ± 1.18 (7.45–7.81)	8.04 ± 1.23 (7.85–8.23)	8.78 ± 1.39 (8.57–9.00)	<0.001* [a,b: 0.199, Other pairs: <0.05]
DAR	4.21 ± 0.60 (4.11–4.30)	4.24 ± 0.57 (4.15–4.33)	4.37 ± 0.59 (4.28–4.63)	4.42 ± 0.67 (4.32–4.52)	0.004* [a,c:0.013, a,d: 0.003, b,c: 0.038, b,d: 0.008]
**ORA parameters**					
CH (mmHg)	10.71 ± 2.24 (10.35–11.07)	10.32 ± 2.13 (9.97–10.66)	10.20 ± 2.27 (9.83–10.57)	10.42 ± 3.35 (9.88–10.96)	0.24
CRF (mmHg)	10.40 ± 2.02 (10.06–10.74)	10.29 ± 1.93 (9.70–10.11)	10.35 ± 2.06 (10.00–10.67)	10.78 ± 4.38 (10.04–11.51)	0.693

Significant results are represented with*.

*(n = 172 eyes) (SD, Standard Deviation; CI, Confidence Interval; AL, Applanation Length; AV, Applanation Velocity; HCR, Highest Concavity Radius; DA, Deformation Amplitude; PD, Peak Distance; SP–A1, Stiffness Parameter at First Applanation; DAR, Deformation Amplitude Ratio; IR, Integrated Radius; CH, Corneal Hysteresis; CRF, Corneal Resistance Factor)*.

The pressure-derived parameters of ORA, CH (*p* = 0.240) and CRF (*p* = 0.839), did not show a significant difference among the different myopia groups.

The results of multiple regression analyses to assess the relationship between corneal biomechanical parameters with SE, AXL, bIOP, mean KR, and CCT were shown in Table 3.

**Table 3:** Multiple regression analysis for variables predicting corneal biomechanical parameters.

**Variables**	**Predictors**	**Unstandardized Coefficient B**	**Standardized Coefficient Beta**	***P*–value**	**Adjusted *R*^**2**^**
AL1	CCT	0.003 (0.001–0.005)	0.322	0.005	0.070
	bIOP	0.051 (0.004–0.099)	0.237	0.035	
AL2	CCT	0.004 (0.001–0.006)	0.285	0.005	0.072
AV1	CCT	0.000 (−0.011–−0.008)	−0.460	<0.001	0.506
	bIOP	−0.010 (0.001–0.005)	−0.817	<0.001	
	**SE**	**−0.002** **(−0.0031 to 0.000)**	**−0.179**	**0.016**	
AV2	bIOP	0.016 (0.003–0.028)	0.280	0.013	0.085
	CCT	0.000 (0.000–0.001)	0.172	0.123	
	**AXL**	**−0.022** **(−0.041 to** **−0.003)**	**−0.225**	**0.024**	
PD	bIOP	−0.130 (−0.175 to −0.086)	−0.559	<0.001	0.299
	CCT	−0.003 (−0.005 to −0.001)	−0.322	<0.001	
	**AXL**	**0.110** **(0.041–0.179)**	**0.271**	**0.002**	
HCR	CCT	0.007 (0.004–0.011)	0.385	<0.001	0.250
	**SE**	**0.106** **(0.048–0.163)**	**0.322**	**<0.001**	
DA	bIOP	−0.056 (−0.067 to −0.045)	−0.823	<0.001	0.532
	CCT	−0.001 (−0.002 to −0.001)	−0.432	<0.001	
	**SE**	**−0.014** **(−0.022 to−0.007)**	**−0.279**	**<0.001**	
SPA1	bIOP	9.011 (6.571–11.451)	0.660	<0.001	0.406
	CCT	0.348 (0.242–0.454)	0.580	<0.001	
	**SE**	**2.035** **(0.390–3.680)**	**0.196**	**0.016**	
IR	CCT	−0.013 (−0.017 to −0.010)	−0.629	<0.001	0.399
	bIOP	−0.249 (−0.338 to −0.160)	−0.511	<0.001	
	**SE**	**−0.108** **(−0.169 to−0.048)**	**−0.293**	**<0.001**	
	Mean KR	0.089 (0.001–0.176)	0.167	0.048	
DAR	CCT	−0.009 (−0.011 to −0.007)	−0.751	<0.001	0.505
	bIOP	−0.148 (−0.191 to −0.104)	−0.560	<0.001	
	Mean KR	0.104 (0.062–0.146)	0.363	<0.001	
CH	CCT	0.015 (0.008–0.022)	0.388	<0.001	0.243
	**SE**	**0.259** **(0.087–0.432)**	**0.274**	**0.004**	
	Mean KR	0.149 (0.023–0.274)	0.216	0.021	
CRF	CCT	0.023 (0.016–0.030)	0.600	<0.001	0.357
	bIOP	0.335 (0.170–0.499)	0.385	<0.001	
	Mean KR	0.192 (0.032–0.352)	0.202	0.019	

*(n = 172 eyes) (AL, Applanation Length; AV, Applanation Velocity; HCR, Highest Concavity Radius; DA, Deformation Amplitude; PD, Peak Distance; SP–A1, Stiffness Parameter at First Applanation; DAR, Deformation Amplitude Ratio; IR, Integrated Radius; CH, Corneal Hysteresis; CRF, Corneal Resistance Factor; SE, Spherical Equivalent; AXL, Axial Length; KR, Keratometry Reading; bIOP, Biomechanically corrected IOP; CCT, Central Corneal Thickness)*.

In multiple regression analysis, while both CCT and bIOP were shown as predictors for many of the biomechanical parameters, only one of the parameters directly related to level of myopia (SE or AXL) appeared with each biomechanical parameter. Only AL1 and AL2 have no relationship with a parameter of myopia in the multiple regressions. Other than AL1 and AL2, these results show progressive reduction in the corneal stiffness with increasing myopia, independent of IOP and CCT. Higher levels of myopia are associated with increased corneal velocity during the first and second applanation phases; increased distance between corneal peaks (PD), decreased corneal radius of curvature (HCR), increased axial corneal displacement or deformation (DA) at the concavity phase; increased integrated inverse radius (IR), and reduced corneal stiffness at the first applanation (SPA1). The Corvis ST parameters showing a significant correlation with SE were AV1 (B coefficient = −0.002, *p* = 0.016), HCR (B coefficient = 0.322, *p* < 0.001), DA (B coefficient = −0.014, *p* < 0.001), SP-A1 (B coefficient= 2.035, *p* = 0.016), and IR (B coefficient = −0.108, *p* < 0.001), and also CH (B coefficient = 0.259, *p* = 0.004) using ORA. In other words, for each extra diopter in myopic SE, there was a 0.002 m/s increase in AV1, a 0.322 mm decrease in the radius of corneal curvature in HC phase, a 0.014 mm increase in DA, a 2.035 mmHg/mm decrease in SPA1, a 0.018 mm^−1^ increase in IR and a 0.259 mmHg decrease in CH. Only parameters showing significant relationship with AXL were AV2 and PD.

**Figure 1:**
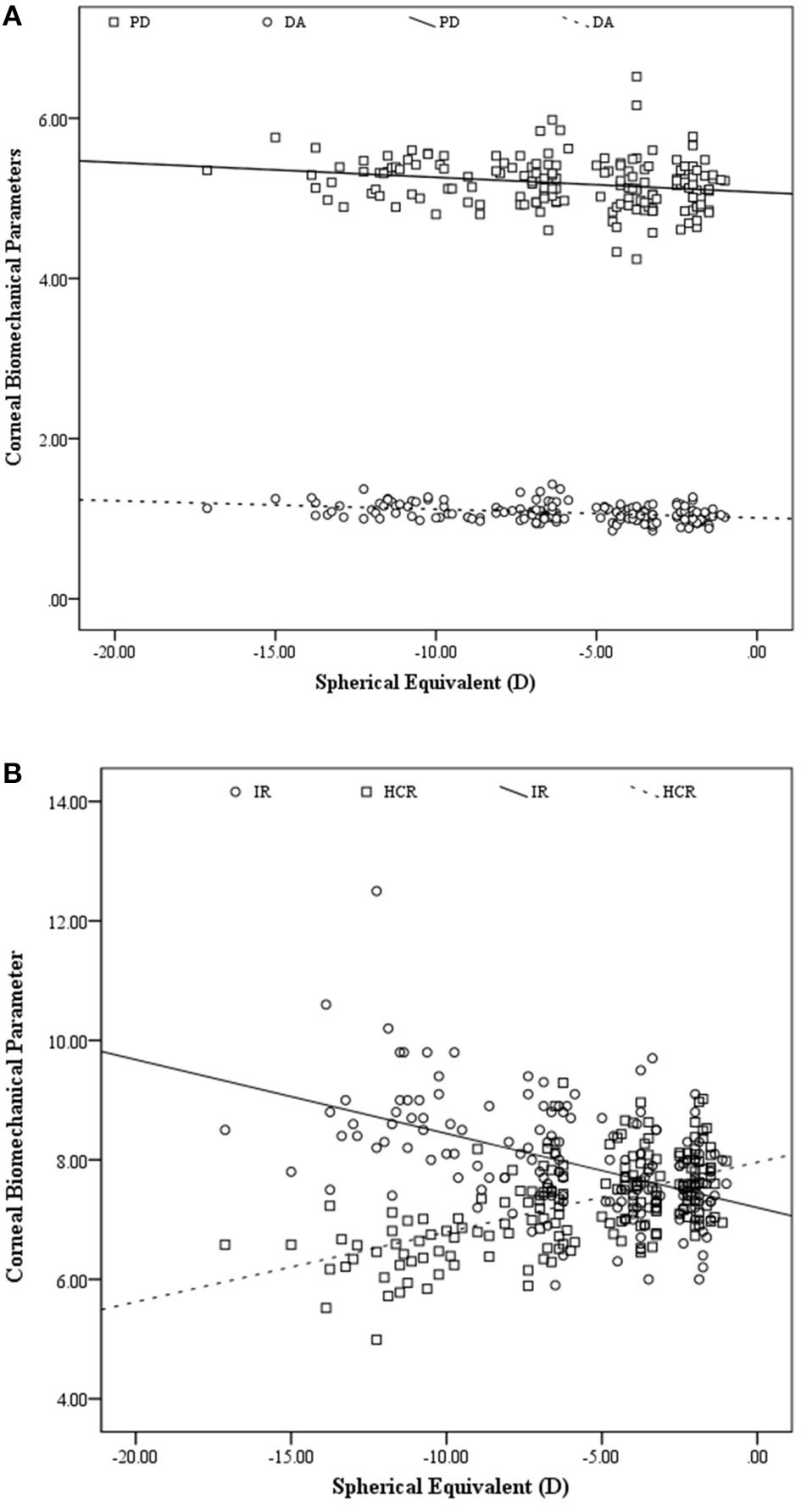
Scatter plot of peak distance (PD) and deformation amplitude (DA) **(A)**highest concavity radius (HCR), and integrated radius (IR) **(B)**as a function of spherical equivalents (*n* = 254 eyes).

A scatter plot of changes in corneal biomechanical parameters as a function of spherical equivalent is presented in Figure 1.

A stepwise comparison was performed to predict level of myopia using biomechanical parameters obtained using Corvis ST and ORA as independent factors showed the HCR (B & beta coefficients = 2.463, 0.553, *p* < 0.001 & Adjusted R square: 0.301) as the strongest predictor of the myopia level. Repeating the analysis using only Corvis ST's parameters as predictors showed a significant relationship for SE with HCR (B coefficient = 2.630, *p* < 0.001), DA(B coefficient = −8.892, *p* = 0.001), and AV1 (B coefficient = 25.947, *p* = 0.009). The adjusted R square for this model was 0.394, so that 39.4% of changes in myopia were predicable based on the predictors remained in this model. Standardized beta coefficients for the predictors were 0.513, −0.241, and 0.172, respectively. It showed that each unit decrease in HCR has more considerable effect on the myopia level.

## Discussion

The current study analyzed the effects of varying degrees of myopia on corneal biomechanical properties using non-contact tonometers which measure corneal deformation and has been shown to have acceptable repeatability (Hon and Lam, 2013; Ye et al., 2015; Lopes et al., 2017). This study is novel in compared to others conducting corneal biomechanical assessment in myopia in that we also assessed patients with extreme myopia.

Our results suggest that among the corneal biomechanical parameters assessed by Corvis ST and ORA, the parameters related to the highest concavity (HC) phase during the assessment by Corvis ST demonstrate not only a significant difference with increasing levels of myopia, but also the strongest correlations. It has been shown that the sclera influences corneal deformation via displaced fluid, and therefore scleral influence is maximum at highest concavity (Nguyen et al., 2018, 2020).

The precise mechanism of structural changes of the eye leading to increasing levels of myopia in childhood and adolescence is not well-understood but previous reports have shown that axial length changes in myopia are associated with changes in corneal structure (Bueno-Gimeno et al., 2014). The biomechanical behavior of the cornea is affected by several factors such as age, IOP, CCT, corneal hydration status, corneal tissue composition, and several other factors, some of which are still under investigation (McMonnies, 2012; Roberts, 2014). The results of this study demonstrate a positive correlation between CCT, mean keratometry, and IOPnct with both CH and CRF, while AXL showed a negative correction only with CH, in keeping with previous studies (Wong and Lam, 2015; Qiu et al., 2016; Wan et al., 2018).

No significant correlation was found between SE with CRF while a positive correlation was seen for CH with SE. This finding is in in agreement with the results of previous studies which have reported a decrease in CH alone with increasing levels of myopia (Qiu et al., 2016; Inceoglu et al., 2018), or a decrease in CH in high myopia (Wu et al., 2019). Also, Shen and colleagues in comparison of ORA's parameters in high myopia (>9.00 D) with a control group with a SE between zero to−3.0 D reported lower CH values in the myopia group (Shen et al., 2008). This is interesting with the different age group of patients recruited (20–35 in this study vs. 11–63 years) and subject selection as subjects were matched subjects upon CCT, IOP, and mean keratometry in the present study.

The results of this study suggest that CRF does not significantly change with increasing levels of myopia, while CH showed a significant decreasing trend generally; same as Shen et al. who reported a decrease in CH with increasing levels of myopia (Shen et al., 2008). Presence of a negative correlation between CH and axial length has been reported in a number of previous studies (Song et al., 2008; Bueno-Gimeno et al., 2014) but is not universal as Lim et al. did not observe any relationship between axial length and CH (Elsheikh et al., 2010). The difference can be attributed to the difference in the subjects' age and race between the two studies.

Whilst a previous study suggested that CH as corneal biomechanical parameter can predict myopia progression in children (Wan et al., 2018); the results of the current study suggest that although there are no significant differences in CH in different myopia groups, there is a positive correlation between CH with SE overall. However, the present patient cohort only included adults and the results cannot be extrapolated to children. In the study by Wan et al. CH was only associated with axial elongation in children using spectacles and not Ortho-K prompting the authors to speculate that CH may be a risk factor for axial elongation in young children not undergoing myopia control. However a number of studies have reported a negative correlation between age and CH/CRF (Kida et al., 2008; Sharifipour et al., 2016) and in the study by Wan et al. a significant difference in baseline and final CH values was also reported. This may explain the lack of a significant difference in CH values in our patient cohort.

However, other biomechanical parameters indicating stiffness, such as those related to the HC corneal phase by Corvis ST, might also be predictive. Future studies should consider the predictive value of the classic and new Corvis ST parameters in determining axial elongation in children with myopia.

Wang et al. in the assessment of parameters obtained by the Corvis ST in 82 subjects aged 21–50 years reported statistically significant differences in DA and HCR between high and moderate myopia groups (Wang et al., 2015). We were able to show differences in corneal biomechanics in patients in comparison between multiple myopic groups.

Comparison of other optical parameters of the eye, assessed in the present study showed no significant difference in the corneal curvature and thickness between the four groups suggesting that the key determinant of the refractive status in the different groups was AXL. Therefore, the current findings did not agree with previous reports that longer eyes have flatter and thinner corneas (Chang et al., 2001). On the other hand, when considering the possible correlation between biomechanics of the cornea and the globe, as well as the evidence of associated thinner sclera and choroidal structures in the larger eyes (Shen et al., 2016) it seems that the elongation of the eye may point to the presence of an abnormal biomechanical behavior of the globe without a clear cause-effect relationship between these factors (Song et al., 2008; Xu et al., 2010).

The findings of the current study with Corvis ST, especially in the HC phase parameters highlighted the biomechanical changes in the group with extreme myopia. The positive correlation between SE with HCR and negative correlation with IR, indicates a “softer” cornea in cases with very high degrees of myopia with longer AXL. In support of this possible conclusion, a recent study reported lower corneal tangent modulus and consequently less corneal stiffness in patients with high levels of myopia (Hon et al., 2017).

Considering SE, the highest correlation with corneal biomechanical parameters was seen for HCR, suggesting that HCR increases as SE approaches zero. This finding may be related to changes occurring in corneal stiffness and consequently the entire globe with a considerable increase in axial length (Chang et al., 2010; Hon et al., 2017) and is in keeping with the findings of a previous study by Wang et al. (2015). There are a number of possible explanations regarding why biomechanical properties of the cornea are correlated to the degree of myopia. Axial elongation has previously been shown to be associated with corneal flattening and thinning cornea, which can lead to changes in corneal biomechanical properties (Chang et al., 2001). Moreover, myopic eyes have a lower level of ocular rigidity compared to their emmetropic and hyperopic counterparts (Lam et al., 2002; Berisha et al., 2010). Finally, myopia progression is associated with significant reduction in the diameter of the scleral collagen fibrils (Phillips and McBrien, 1995; McBrien et al., 2001) a lower level of collagen content and proteoglycan synthesis which ultimately results in scleral thinning and weakening (Rada et al., 2000; McBrien and Gentle, 2003; He et al., 2017). The unique changes in scleral composition of myopes may well-translate into measurable differences in corneal viscoelastic properties and an altered biomechanical response as detected by the ORA and Corvis ST devices.

Of all the parameters evaluated, the two parameters with the highest mean difference between extreme myopia and low myopia groups were HCR and IR, and these parameters also had the high correlation with SE. IR or integrated inverse concave radius is calculated according to the integrated area under the curve of the inverse radius of curvature. A lower IR value points to a stiffer cornea (Fernandez et al., 2017) and it is possible that in patients with extreme myopia, changes in the arrangement of scleral collagen fibers due to axial elongation generates an influence on the corneal mechanical strength and consequently on the scleral stiffness based on the structural similarity of these two tissues (Harper and Summers, 2015). It has been shown that a stiffer sclera will limit corneal deformation. Conversely, a more compliant or softer sclera that is proposed to exist in high and extreme myopia may allow greater displacement of the cornea at highest concavity (Nguyen et al., 2018, 2020).

Our study has a number of limitations. There were a smaller number of eyes included with extreme myopia compared to other groups. Another weakness was the lack of analysis of the ORA signals to include the extracted waveform parameters for comparison and statistical analysis. Furthermore, only adult subjects were recruited and the study's findings cannot be extrapolated to children.

In conclusion, the results of this study suggest that in extreme myopia of more than 9.00 D, corneal biomechanical parameters assessed in the highest concavity (HC) phase by Corvis ST demonstrate a shift in a weaker direction biomechanically, whilst the pressure-derived parameters of ORA showed no significant difference between the groups, although there is a positive correlation between CH and SE overall, indicating reduction of ability to dissipate energy accompanies reduction in stiffness as level of myopia increases. Future studies including analysis of corneal biomechanical properties in children with a family history of extreme myopia or children with progressive myopia may be appropriate for planning purposes and prevention programs.

## Data Availability Statement

The original contributions presented in the study are included in the article/supplementary material, further inquiries can be directed to the corresponding author/s.

## Ethics Statement

The studies involving human participants were reviewed and approved by Ethics Committee of Mashhad University of Medical Sciences, Mashhad, Iran. The patients/participants provided their written informed consent to participate in this study.

## Author Contributions

ZD, CR, NM, and AJ corrected the manuscript. All authors revised the manuscript for significant intellectual content and gave final approval of the version to be published.

## Conflict of Interest

CR is a consultant to Oculus Optikgeräte GmbH, Zeimer Ophthalmic Systems AG, and Optimo Medical. The remaining authors declare that the research was conducted in the absence of any commercial or financial relationships that could be construed as a potential conflict of interest.
